# Security of Semi-Device-Independent Random Number Expansion Protocols

**DOI:** 10.1038/srep15543

**Published:** 2015-10-27

**Authors:** Dan-Dan Li, Qiao-Yan Wen, Yu-Kun Wang, Yu-Qian Zhou, Fei Gao

**Affiliations:** 1 State Key Laboratory of Networking and Switching Technology, Beijing University of Posts and Telecommu-nications, Beijing, 100876, China; 2 State Key Laboratory of Cryptology, P. O. Box 5159, Beijing, 100878, China

## Abstract

Semi-device-independent random number expansion (SDI-RNE) protocols require some truly random numbers to generate fresh ones, with making no assumptions on the internal working of quantum devices except for the dimension of the Hilbert space. The generated randomness is certified by non-classical correlation in the prepare-and-measure test. Until now, the analytical relations between the amount of the generated randomness and the degree of non-classical correlation, which are crucial for evaluating the security of SDI-RNE protocols, are not clear under both the ideal condition and the practical one. In the paper, first, we give the analytical relation between the above two factors under the ideal condition. As well, we derive the analytical relation under the practical conditions, where devices’ behavior is not independent and identical in each round and there exists deviation in estimating the non-classical behavior of devices. Furthermore, we choose a different randomness extractor (i.e., two-universal random function) and give the security proof.

Truly random numbers have been wildly applied in many aspects such as numerical simulations of physical and biological systems, gambling and cryptography. As we know, the security of quantum key distribution (QKD) protocols depends on random selections of the prepared states and measurements so that adversary cannot utilize an attack to get secret information without being discovered.

There is no intrinsic randomness in the world of classical physics. In principle, any classical system admits a perfect description. And any observed randomness of a classical process is apparent (called as apparent randomness^1^), since it can be explained as the probabilistic mixture of deterministic classical events. Specially, the existing random number generators such as the linear feedback shift registers, which are characterized by using the deterministic algorithms, generate apparent randomness for us due to lacking of knowledge about their precise descriptions.

The advent of quantum physics makes it possible to produce intrinsic randomness. Colbeck *et al.*^2^ gave a RNE protocol based on Greenberger-Horne-Zeilinger (GHZ) paradox. Pironio *et al.*^3^ proposed a RNE protocol, where the generated randomness was certified by non-local correlation in the Clauser-Horn-Shimony-Holt (CHSH) test and quantified by min-entropy^4^,^5^,^6^ of measurement outcomes. Fehr *et al.*^7^ further characterized the amount of the generated randomness based on the ref. 3 and proposed a superpolynomial RNE protocol. Pironio *et al.*^8^ analyzed that honest and dishonest device suppliers had influence on RNE and optimized conclusions of the ref. 3. The above protocols are categorized as DI-RNE ones, which make no assumption about the internal working of the devices.

As is well-known, DI-RNE protocols require entanglement, which results in negative effects on the complexity of devices and the rate of randomness generation. Thus the question whether we can generate randomness without any entanglement may arise. Fortunately, Li *et al.*^9^ proposed SDI-RNE protocols without entanglement based on 2 → 1 quantum random access code (QRAC)^10^,^11^ and the generated randomness was certified by non-classical correlation in the prepare-and-measure test. Furthermore, Li *et al.*^12^ generalized the case of the ref. 9 to more general ones (i.e., *n* → 1 QRAC) and pointed out 3 → 1 QRAC was the most efficient SDI-RNE protocols. These SDI-RNE protocols, where the users have no knowledge of internal working of the devices except for the dimension of the systems, are preferred since they are convenient for application.

The security of RNE protocols is of importance. As the security of QKD protocols^13^,^14^,^15^,^16^ emphasizes key rate, the security of RNE ones focuses on the amount of the generated randomness. In the above mentioned DI-RNE protocols, the analytical relations between the amount of the generated randomness and Bell inequality violation was presented under the ideal and practical conditions^3^,^7^,^8^. And in the SDI scenario, the relation between the amount of the generated randomness and the degree of non-classical correlation was given by using Levenberg-Marquadrt (L-M) algorithm^9^,^12^ and semi-definite programm (SDP) relaxation^17^,^18^,^19^ under the ideal condition, respectively.

There are some problems worth thinking about in the SDI-RNE protocols. The analytical relation between the amount of the generated randomness and the degree of non-classical correlation under the ideal condition is missing. In practice, the behavior of the device is not identical and independent in each round and there exists deviation in estimating the non-classical behavior of the devices. It is natural to ask that the amount of the generated randomness and the degree of non-classical correlation satisfy what kind of analytical relation considering the above practical conditions.

In the paper, we give the analytical relation between the amount of the generated randomness and the degree of non-classical correlation under the ideal condition. Furthermore, we consider the practical conditions and establish the analytical relation which is described by a lower bound on the amount of the generated randomness based on the non-classical behavior of the devices. Finally, we choose two-universal random function^20^ as randomness extractor and give the security proof.

## Results

### The model of SDI-RNE protocols^12^

Suppose that the relevant dimension *d* of the quantum systems are- known, in this work we take *d* = 2. But the prepared states and measurement are not described. Generally, Alice’s and Bob’s black boxes are systems for state preparation 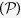 and measurement 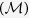. Alice chooses *n* bits *x* = *x*_0_*x*_1_... *x*_*n*−1_ ∈ {0, 1}^*n*^ at random, and sends the encoded state 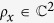 to Bob. Then Bob chooses a measurement operator 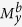 acting on the state *ρ*_*x*_ with input parameter *y* ∈ {0, 1,..., *n* − 1} and output parameter *b* ∈ {0, 1}, where 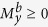, 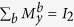. After repeating the procedure infinite times, Alice and Bob can get the probability distribution 
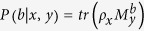
. The generated randomness can be certified by the non-classical correlation.

Denote


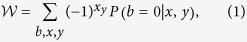


called as 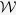 *expression*. If the systems admit a classical description, then 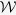 expression based on 2 → 1 QRAC satisfies 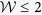, denoted as 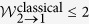 simply. Obviously, if the systems contain the non-classical correlation (i.e., certain measurements act on quantum states), the data can violate the above inequality and makes 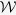 expression value up to 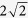 
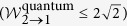
. Similarly, 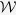 expression based on 3 → 1 QRAC satisfy 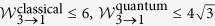.

The amount of randomness of output *b* conditioned on the inputs *x, y* can be characterized by the *min-entropy*^4^


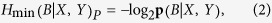


where the *maximal guessing probability*^4^ of *B* given *X, Y* is


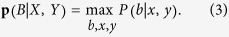


Based on equation (2), exploring a lower bound on min-entropy is equivalent to the upper bound on maximal guessing probability. So, to calculate the amount of the generated randomness can be converted into exploring maximal guessing probability for given value of 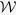 expression in the following optimization problem.


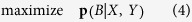


subject to:


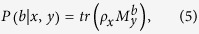



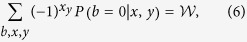


where the optimization is carried out by arbitrary quantum state *ρ*_*x*_ and positive operator valued measure (POVM) 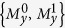 defined over two dimensional Hilbert space.

#### Analytical relation under the ideal condition

We give the analytical relation between the maximal guessing probability and the corresponding maximal value of 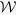 expression. Moreover, we get the explicit bounds of 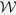 expression when there is the generated randomness. In other words, we gain the reason why there is not the generated randomness when the data just violates the classical bound of 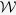 expression. Here, we mainly give the results of the primitive ones (proved in the Supplementary Information).

Theorem 1. *Suppose that SDI-RNE protocol based on* 2 → 1 *QRAC is associated with two dimensional Hilbert space. The analytical relation between the maximal guessing probability* **p** *and the corresponding maximal value of* 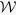 *expression is given as*


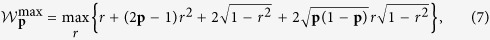


*where* 
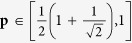
 *and r is one of the real roots of equation* (8) *with a variable x*


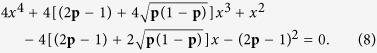


According to the analytical relation (7), denoted as 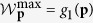, we explore the critical value of 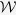 expression conditioned on there exists the generated randomness. Let **p** = 1 (i.e., there is not the generated randomness of the outputs), we get 
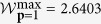
 (*r* = 0.7904) by taking over all the real roots of the equation expressed as 4*x*^4^ + 4*x*^3^ + *x*^2^ − 4*x* − 1 = 0. Further, we learn that *g*_1_ is the monotonically decreasing and continuous function. As long as 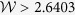, the outputs exhibit randomness (**p** < 1).

Theorem 2. *Suppose that SDI-RNE protocol based on* 3 → 1 *QRAC is associated with two dimensional Hilbert space. The analytical relation between the maximal guessing probability* **p** *and the corresponding maximal value of* 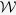 *expression is given as*


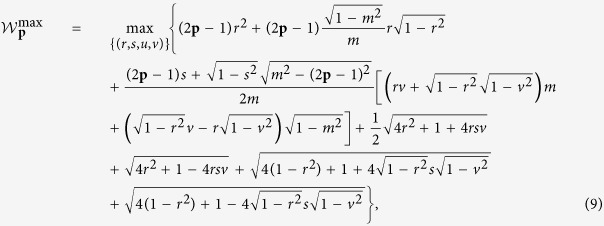


*where* 
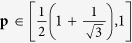
 *and the values of* (*r, s, v, m*) *is one of the real roots of the equation set in variables* (*x, y, z, u*) *in the* Supplementary Information.

Similar to the above analysis, we calculate the critical value of 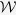 expression conditioned on there exists the generated randomness. Let **p** = 1, we get 
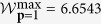
 ((*r, s, v, m*) = (0.7730, 0.3837, −0.1529, 1)) by taking over all the real roots of the equation set in the Supplementary Information. So, we conclude that as long as 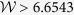, the generated randomness can be certified.

#### Analytical relation under the practical condition

In practice, there exist some unideal factors during the experiment, for example, the behavior of the devices is not identical and independent in each round, and estimating the non-classical behavior of the devices causes deviation. We establish the analytical relation between the amount of the generated randomness and the degree of non-classical correlation under the practical condition. As well, our result can be applied to any RNE protocols with quantum system of arbitrary dimension and a general form of 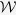 expression in the SDI scenario.

### Description of the devices used *t* times in succession

We consider a pair of devices 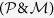, where the state preparation 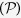 and measurement 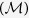 can be regarded as two black boxes. The preparation box contains a set of arbitrary states 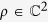 and the measurement box contains a sequence of arbitrary measurements 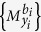 defined over two-dimensional Hilbert space, where measurement operator 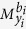 represents input parameter *y*_*i*_ and output parameter *b*_*i*_.

We make the most basic assumptions as follows:

1. the preparation system and the measurement system conform to the quantum theory;
2. there is no additional communication between the state preparation system and the measurement system in each round. That is, the state preparation system and the measurement system have a single qubit for communication and are not allowed to divulge information to eavesdropper in each round;
3. the inputs *X, Y* are random variables that are independent and uncorrelated with the devices.

No constrains are imposed on the states and measurements except for their dimension and the above assumptions. But the behavior of devices is not identical and independent in each round *i*, which implies that the previous *i* − 1 states, measurement operators and measurement outcomes affect the *i*th measurement outcomes. Note that we assume that the state preparation system are not entangled with the measurement system or any other party in the following calculation of the amount of generated randomness, which is similar to that in previous work^7^,^8^.

We denote the inputs by *x*_*i*_ ∈ **X**, *y*_*i*_ ∈ **Y** and the measurement output by *b*_*i*_ ∈ **B** in the *i*th round. We denote the first *i* inputs by *x*^*i*^ = (*x*_1_, *x*_2_,..., *x*_*i*_) and define *y*^*i*^, *b*^*i*^ similarly. The devices’ behavior cannot be identical and independent in each round. That is, the behavior of devices varies from one round to another making use of internal memory, which is depicted by a sequence of unitrary transformations *U*_0_,..., *U*_*t*−1_ acting on 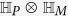. *U*_*i*−1_ is used for the state and the measurement operator before the *i*th round (*U*_0_ = *I* in the first round). In details, suppose that Alice chooses the state 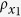 at will and Bob chooses the measurement setting 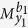 in the first round, we get 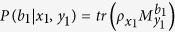. Alice and Bob choose 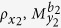 at random, due to un-identical and dependent between rounds, we get 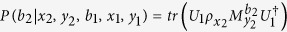, where the operation *U*_1_ encodes the information of the inputs *x*_1_, *y*_1_ and output *b*_1_ in the first round. The given conditional probability distribution 
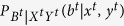
, which describes the input-output behavior of *t* sequential interactions with the devices 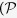 & 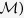, is defined as


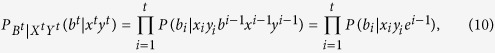


where 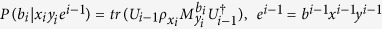. The first equality holds because of successive Bayes’ principle and the second one shows that the output in the *i*th round is determined by the inputs of the *i*th round and the pervious inputs and outputs.

We learn that there is one-to-one correspondence between the maximal guessing probability and the corresponding maximal value of 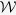 expression based on the analytical relations (i.e., collectively called *g*_1_) in the above part. The analytical relations show


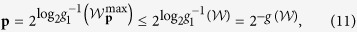


where *g*_1_ is the monotonically decreasing and continuous function of the corresponding maximal value of 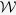 and 
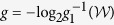
 is the convex function of the value of 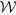 expression.

### Estimating the degree of non-classical correlation

Here, we estimate 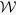 expression value to characterize the degree of non-classical correlation.

For the first round, 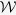 expression value is established by 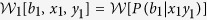. For other rounds, there are slightly different because of the present round depending on the inputs and outputs of the previous rounds. So, 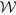 expression value in the *i*th round is 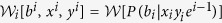.

Let


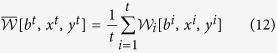


be the average value of 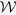 expression, averaged over *t* rounds. In order to estimate the average value 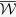, we introduce the following estimator 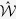, determined from the observed statistics:


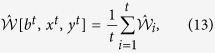


where 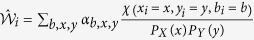 is the observed value of 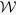 expression in the *i*th round and *χ*(*x*) is the indictor function:


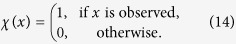


We derive the result of estimating the average value 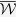 in the following (proved in the Supplementary Information).

Lemma 3. *Let the symbols be the same as before. For any δ* > 0*, the average value* 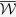 *and the observed average value* 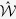 *satisfy*


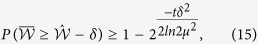


*where* 
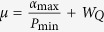
, *α*_max_ = max|{*α*_*b*,*x*,*y*_}|, *P*_min_ = min{*P*(*x*)*P*(*y*)} *and W*_*Q*_ *is the maximal value of* 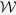 *expression allowed by quantum theory*.

From inequality (15), we learn that the average value 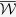 can be larger than the observed average value 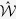 up to some *δ* with probability 1 when experiment’s rounds tend toward infinity.

### Bounding the min-entropy

Here, we proceed with the last step to get the analytical relation between the amount of the generated randomness and the observed average value 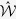 under the practical conditions. Just as the refs 7, 8 consider the average Bell value in some interval as a prior condition to make the min-entropy meaningful in the DI case, we use the technique^7^ to quantify the generated randomness, which is depicted by a lower bound on min-entropy of outputs conditioned on the event that the observed average value 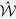 lies in some interval.

Denote *W*_0_ by the maximal value of 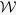 expression conditioned on *H*_min_(*B*^*t*^|*X*^*t*^*Y*^*t*^) = 0. *W*_0_ > *W*_*cl*_ (the classical bound of 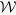 expression), which is different from that of Bell experiments. We partition the interval [*W*_0_, *W*_*Q*_] ⊂ *R* into 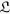 disjoint blocks: 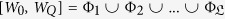 with Φ_*l*_ = [*W*_*l*−1_, *W*_*l*_).

Here, a basic event space 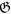 is the set that includes all possible (*b*^*t*^, *x*^*t*^, *y*^*t*^, *l*) for the above experiment. Define an event 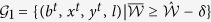. According to Lemma 3, the event 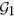 occurs with high probability. In fact, the values of (*b*^*t*^, *x*^*t*^, *y*^*t*^) can determine the value of 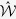 and random variable *l*. Next, we define an event 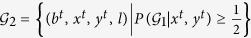 and an event 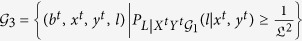. Let 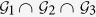 be the good event, denoted as 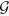. We call 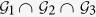 as the good event (i.e., 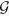) since we can get the amount of the generated randomness as long as all of the events 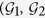, and 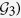 occur. Note that an event is a set that contains one or more results of a basic event space, which is a subset of the basic event space. As well, each result of an event is a element (basic event).

The following lemma is proven in the Supplementary Information.

Lemma 4. *There exist the above good event* 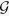 *with probability*


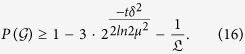


We try to put a bound on the min-entropy of the outputs *B*^*t*^ conditioned on the inputs (*X*^*t*^, *Y*^*t*^) and the observed average value 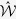 in some interval.

Theorem 5. *Let* (*X, Y*) *be identical, independent and random sources and δ* > 0 *be an arbitrary parameter. For any devices’ behavior, the observed distribution P* = {*P*(*b*^*t*^, *x*^*t*^, *y*^*t*^)} *characterizing successive t rounds satisfies*


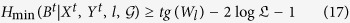


*for all*
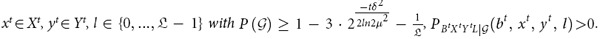


*Proof.* Without loss of generality, suppose that *l* is the unique value with 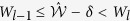.

Let 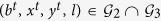, we consider nontrivial cases, i.e., 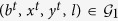. Otherwise, 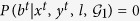.

According to the description of 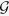, we get


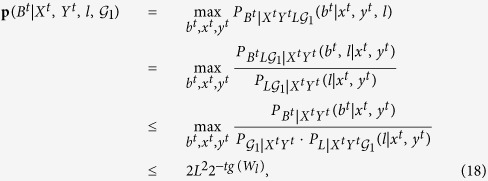


where the penultimate inequality holds because of 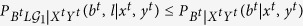 and the last one holds by using equations (10), (11) and (12).

Furthermore, with the above inequality, it is easy to show that


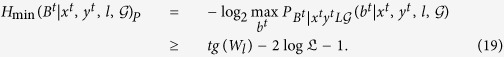


**Figure 1:**
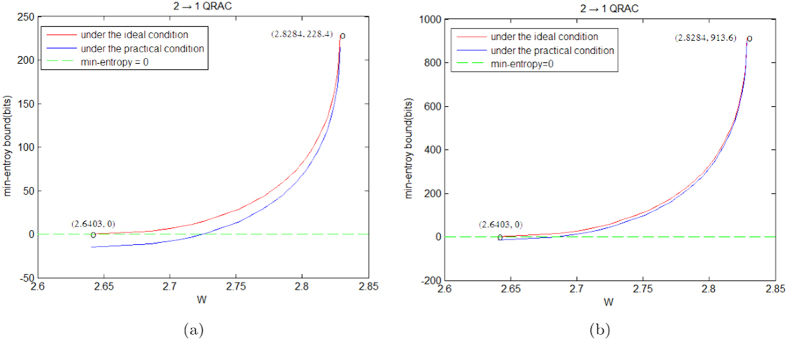
Compare the lower bound on the amount of the generated randomness in the SDI-RNE protocol based on 2 → 1 QRAC under the different conditions. (**a**) Under the condition of the experiment’s rounds *t* = 1000. (**b**) Under the condition of the experiment’s rounds *t* = 4000.

**Figure 2:**
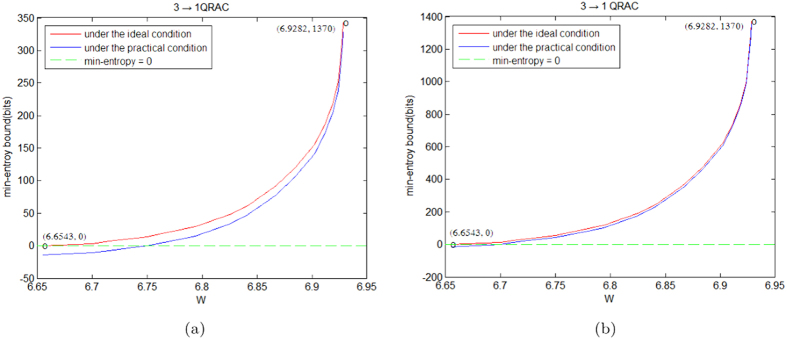
Compare the lower bound on the amount of the generated randomness in the SDI-RNE protocol based on 3 → 1 QRAC under the different conditions. (**a**) Under the condition of the experiment’s rounds *t* = 1000. (**b**) Under the condition of the experiment’s rounds *t* = 4000.

Here, suppose that disjoint blocks 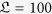, *δ* = 0.0001 and the experiment’s rounds *t* = 1000, 4000, respectively. Under the ideal and practical conditions, we compare the lower bound on min-entropy of the generated randomness of SDI-RNE protocols based on 2 → 1 and 3 → 1 QRACs in Figs 1 and 2, respectively. Obviously, when rounds of experiments is increasing and the number of the disjoint blocks is fixed, the Figures reveal that the gap of the amount of the generated randomness between the ideal and practical conditions is rapidly closing. Note that *W* in the Figures represents the observed average value.

#### Randomness extraction

As we know, by using a randomness extractor^20^,^21^, the outputs *b*^*t*^ can be converted to a string that is nearly uniform and uncorrelated to the information of an adversary.

We propose a SDI-RNE protocol with another randomness extractor which is different from ones of the refs 7, 8. The users ask providers for two devices, where state preparation 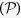 has 2^*n*^ settings and measurement 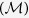 has *n* settings and can make two possible output 0, 1. Furthermore, the users ask that these devices satisfy the most basic assumptions. But, they have no knowledge of the internal working of devices except for their dimension. The protocol is presented in the following.

The users allow a single qubit to communicate in each round and do not send any information outside the laboratory.

1. Divide their initial truly random string 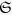 into *S*_1_ and *S*.
2. Introduce (*x*_*i*_, *y*_*i*_) ∈ *S*_1_ into the devices and obtain output *b*_*i*_.
3. Repeat step (2) until exhausting *S*_1_ and build a output string.
4. Calculate the observed average value and determine the value *l* that 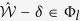. If 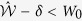, the protocol aborts.
5. Make use of *S* to choose the two-universal random function *f* and obtain a finial string. Based on Theorem 5, the length of the finial string is

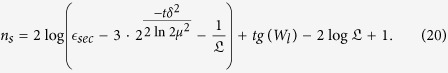


In order to prove security of the proposed protocols, we make the lemma for preparation (proved in the Supplementary Information).

Lemma 6. *Suppose that* 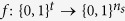 *is the two-universal random function*^22^ *and* 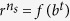
*, where b*^*t*^ ∈ {0, 1}^*t*^*. We get*


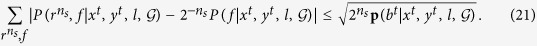


Theorem 7. *The proposed SDI-RNE protocol is* 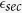 *secure. That is, it is* 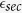 *indistinguishable from a ideal protocol.*

*Proof.* Based on the definition of security of protocol, we get


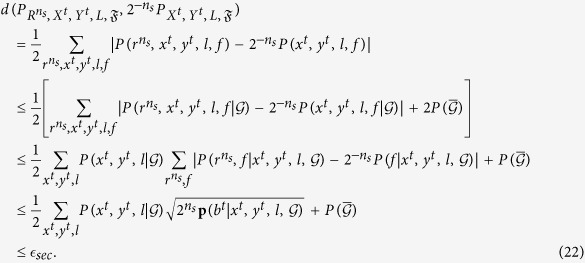


The penultimate inequality holds by using by the above Lemma 6.

## Discussion

In the paper, we have showed the analytical relations between the amount of the generated randomness and the degree of non-classical correlation under the ideal and practical conditions. As a byproduct, the critical values of 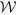 expression have been presented when there exists the generated randomness. Moreover, the case, where the adversary holds the classical side information^8^ of the devices, can be regarded as our case conditioned on the particular value of the side information. Finally, we choose the two-universal function as randomness extraction and give the security proof. Whereas, there are still interesting questions that remain open. How can we quantify the generated randomness by directly using the observed probability distribution. Furthermore, for a given observed probability distribution, whether and how to find an optimal witness of given dimension with the method in the refs 19.

## Additional Information

**How to cite this article**: Li, D.-D. *et al.* Security of Semi-Device-Independent Random Number Expansion Protocols. *Sci. Rep.* **5**, 15543; doi: 10.1038/srep15543 (2015).

## Supplementary Material

Supplementary Information

## References

[b1] Dhara C., De La Torre G. & Acn A. Can observed randomness be certified to be fully intrinsic. Phys. Rev. Lett. 112, 100402 (2014). 24679271 10.1103/PhysRevLett.112.100402

[b2] Colbeck R. & Kent A. Private randomness expansion with untrusted devices. J. Phys. A: Math. Theor. 44, 095305 (2011).

[b3] Pironio S. *et al.* Random numbers certified by Bell’s theorem. Nature (London) 464, 1021 (2010). 20393558 10.1038/nature09008

[b4] Köenig R., Renner R. & Schaffner C. The operational meaning of min and max-entropy. IEEE Trans. Inf. Theory 55, 4337–4347, (2009).

[b5] Tomamichel M., Colbeck R. & Renner R. Duality between smooth min and max-entropies. IEEE Trans. Inf. Theory 56, 4674–4681 (2010).

[b6] Köning R. & Renner R. Sampling of min-entropy relative to quantum konwledge. IEEE Trans. Inf. Theory 57, 4760–4787 (2011).

[b7] Fehr S., Gelles R. & Schaffner C. Security and composability of randomness expansion from Bell inequalities. Phys. Rev. A 87, 012335 (2013).

[b8] Pironio S. & Massar S. Security of practical private randomness generation. Phys. Rev. A 87, 012336 (2013).

[b9] Li H. W. *et al.* Semi-device-independent random-number expansion without entanglement. Phys. Rev. A 84, 034301 (2011).

[b10] Ambainis A., Leung D., Manciska L. & Ozols M. Quantum random access codes with shared randomness. e-print arXiv:quant-ph/0810.2937v3.

[b11] Pawłowski M. & Éukowski M. Entanglement-assisted random access codes. Phys. Rev. A 81, 042326 (2010).

[b12] Li H. W., Pawłowski M., Yin Z. Q., Guo G. C. & Han Z. F. Semi-device-independent randomness certification using *n* → 1 quantum random acess codes. Phys. Rev. A 85, 052308 (2012).

[b13] Acn A. *et al.* Device-independent security of quantum cryprography against collective attacks. Phys. Rev. Lett. 98, 230501 (2007). 17677888 10.1103/PhysRevLett.98.230501

[b14] Christandle M., Renner R. & Ekert A. A generic security proof for quantum key distribution. e-print arXiv: quant-ph/0402131.

[b15] Masanes S., Pironio S. & Acn A. Security device-independent quantum key distribution with causally independent measurement devices. Nature Commun. 2, 238–251 (2011). 21407204 10.1038/ncomms1244

[b16] Masanes L., Renner R., Christandl M., Winter A. & Barrett J. Full security of quantum key distribution from no-signalling contraints. IEEE Trans. Inf. Theory 60, 4973–4986 (2014).

[b17] Li H. W. *et al.* Relation between semi- and fully-device-independent protocols. Phys. Rev. A 87, 020302(R) (2013).

[b18] Mironowicz P., Li H. W. & Pawłowski M. Properties of dimension witnesses and their semidefinite programming relaxations. Phys. Rev. A. 90, 022322 (2014).

[b19] Navascués M. & Vértesi T. Bounding the set of finite dimensional quantum correlations. Phys. Rev. Lett. 115, 020501 (2015). 26207454 10.1103/PhysRevLett.115.020501

[b20] De A., Portmann C., Vidick T. & Renner R. Trevisan’s ertractor in the presence of quantum side information. e-print arXiv: quant-ph/0912.5514v3.

[b21] Aroya A. B. & Shma A. T. Better short-seed quantum-proof extractors. Theoretical Computer Science 419, 17–25 (2012).

[b22] Bennett C. H., Brassard G., Crepeau C. & Maurer U. M. Generalized privacy amplification. IEEE Trans. Inf. Theory 41, 1915–1923 (1995).

