# Supplementary material for: Security of Semi-Device-Independent Random Number Expansion Protocols
Source: Sci Rep. 2015 Oct 27;5:15543. doi: 10.1038/srep15543 (PMC4622094; doi:10.1038/srep15543)
Supplement: Supplementary Information [file srep15543-s1.pdf]

Supplementary information of "security of semi-device-independent random number expansion protocols"

Dan-Dan Li[1,2], Qiao-Yan Wen[1], Yu-Kun Wang[1], Yu-Qian Zhou[1] & Fei Gao[1]

[1] *State Key Laboratory of Networking and Switching Technology, Beijing University of Posts and Telecommunications, Beijing, 100876, China*

[2] *State Key Laboratory of Cryptology, P. O. Box 5159, Beijing, 100878, China*

Analytical formula under the ideal condition.

Proof of Theorem 1. Based on $2 \to 1$ QRAC, let the set of states be $\{\rho_{00}, \rho_{01}, \rho_{10}, \rho_{11}\}$. An arbitrary mixed state can be described as a convex combinations of the pure states. And $\mathcal{W} = \sum_{x,y}(-1)^{xy}P(0|x,y)$ is the linear expression of probabilities. To explore the maximal value of $\mathcal{W}$, we can only consider the pure states in the numerical calculation. As well, Masanes [1] has proven that the POVM can be depicted as a convex combinations of projective measurements in the case of two-measurement outcomes. So, we only consider the projective measurements $\{M_y^0, M_y^1\}$, where $M_y^0 + M_y^1 = I$ for $y = 0, 1$. $\mathcal{W}$ expression can be described as

$$\begin{aligned}\mathcal{W} =& \sum_{x,y}(-1)^{xy}P(0|x,y) = \sum_{x,y}(-1)^{xy}tr(\rho_x M_y^0)\\ =& \frac{1+\overrightarrow{r_{00}}\cdot\overrightarrow{a_0}}{2} + \frac{1+\overrightarrow{r_{00}}\cdot\overrightarrow{a_1}}{2} + \frac{1+\overrightarrow{r_{01}}\cdot\overrightarrow{a_0}}{2} - \frac{1+\overrightarrow{r_{01}}\cdot\overrightarrow{a_1}}{2} - \frac{1+\overrightarrow{r_{01}}\cdot\overrightarrow{a_0}}{2} + \frac{1+\overrightarrow{r_{10}}\cdot\overrightarrow{a_1}}{2} - \frac{1+\overrightarrow{r_{11}}\cdot\overrightarrow{a_0}}{2} - \frac{1+\overrightarrow{r_{11}}\cdot\overrightarrow{a_1}}{2}\\ =& \frac{1}{2}[(\overrightarrow{a_0}+\overrightarrow{a_1})\cdot(\overrightarrow{r_{00}}-\overrightarrow{r_{11}}) + (\overrightarrow{a_0}-\overrightarrow{a_1})\cdot(\overrightarrow{r_{01}}-\overrightarrow{r_{10}})],\end{aligned} \tag{1}$$

where $\overrightarrow{r_x}$, $\overrightarrow{a_y}$ represent the Bloch vectors of the pure states ρ_x and the projective measurement operators M_y^0, respectively.

Suppose that θ, γ_1 are the angles between $\overrightarrow{a_0}$ and $\overrightarrow{a_1}$, $\overrightarrow{r_{01}}$ and $\overrightarrow{r_{10}}$, respectively. By using the parallelogram rule of addition of vectors, we get

$$\overrightarrow{a_0}+\overrightarrow{a_1} = 2\cos\frac{\theta}{2}\overrightarrow{u_1}, \quad \overrightarrow{a_0}-\overrightarrow{a_1} = 2\sin\frac{\theta}{2}\overrightarrow{u_2}, \tag{2}$$

$$\overrightarrow{r_{01}}-\overrightarrow{r_{10}} = 2\sin\frac{\gamma_1}{2}\overrightarrow{v_1}, \tag{3}$$

where $\overrightarrow{u_1}(\overrightarrow{u_2},\overrightarrow{v_1})$ represents the unit vector overlapped perfectly with $\overrightarrow{a_0}+\overrightarrow{a_1}, (\overrightarrow{a_0}-\overrightarrow{a_1}, \overrightarrow{r_{01}}-\overrightarrow{r_{10}})$.

Suppose that the projection of $\overrightarrow{r_{00}}$ is $\overrightarrow{r_{00}}'$ in the plane, which is spanned by $\overrightarrow{a_0}$ and $\overrightarrow{a_1}$. Without loss of generality, $\overrightarrow{r_{00}}'$ lies in the region between $\overrightarrow{a_0}$ and $\overrightarrow{a_1}$. Let α, β be the angles between $\overrightarrow{r_{00}}'$ and $\overrightarrow{a_0}$, $\overrightarrow{r_{00}}'$ and $\overrightarrow{r_{00}}$, equation (1) can be depicted as

$$\mathcal{W} = \frac{1}{2}[\cos\alpha\cos\beta + \cos(\theta-\alpha)\cos\beta - 2\cos\frac{\theta}{2}\overrightarrow{u_1}\cdot\overrightarrow{r_{11}} + 4\sin\frac{\theta}{2}\sin\frac{\gamma_1}{2}\overrightarrow{u_2}\cdot\overrightarrow{v_1}]. \tag{4}$$

In general, let the angle between ρ_{00} and a_0 be the minimum, which indicates that the guessing probability $p(0|00,0)$ is the maximum of the set of $P(b|x,y)$. We obtain $\alpha \in [0, \frac{\pi}{4}]$, $\theta \in [2\alpha, \pi - 2\alpha]$. Fixing the angles of α, θ and applying the equation (4), then we get the maximal value of $\mathcal{W}$ expression

$$\begin{aligned}\mathcal{W}_{\mathbf{p},\theta} =& \frac{1}{2}[\cos\alpha + \cos(\theta-\alpha) + 2\cos\frac{\theta}{2} + 4\sin\frac{\theta}{2}]\\ =& (2\mathbf{p}-1)(\cos\frac{\theta}{2})^2 + 2\sqrt{\mathbf{p}(1-\mathbf{p})}\cos\frac{\theta}{2}\sqrt{1-(\cos\frac{\theta}{2})^2} + \cos\frac{\theta}{2} + 2\sqrt{1-(\cos\frac{\theta}{2})^2},\end{aligned} \tag{5}$$

where $\mathbf{p} = \frac{1+\cos\alpha}{2}$ is the maximal guessing probability and the first equality holds because of setting $\beta = 0, \gamma_1 = \pi, \overrightarrow{r_{11}} = -\overrightarrow{u_1}, \overrightarrow{v_1} = \overrightarrow{u_2}$.

Furthermore, in order to obtain the maximal value of $\mathcal{W}$ expression only about the maximal guessing probability $\mathbf{p}$ (i.e., the angle of α), we use the method of the extreme-value problem of function and let $x = \cos\frac{\theta}{2}$. Applying equation (5), we get

$$\mathcal{W}_{\mathbf{p}}^{\max} = \max_{\{r\}}\{r + (2\mathbf{p}-1)r^2 + 2\sqrt{1-r^2} + 2\sqrt{\mathbf{p}(1-\mathbf{p})}r\sqrt{1-r^2}\}, \tag{6}$$

where r is one of the real roots of $4x^4+4[(2\mathbf{p}-1)+4\sqrt{\mathbf{p}(1-\mathbf{p})}]x^3+x^2-4[(2\mathbf{p}-1)+2\sqrt{\mathbf{p}(1-\mathbf{p})}]x+(2\mathbf{p}-1)^2 = 0$.

Proof of Theorem 2. Based on $3 \to 1$ QRAC, let the set of states be $\{\rho_{000}, \rho_{001}, ..., \rho_{110}, \rho_{111}\}$. An arbitrary mixed state can be described as a convex combinations of the pure states. And Masanes [1] has proven that the POVM can be depicted as a convex combination of projective measurements in the case of two-measurement outcomes, we only consider projective measurements $\{M_j^0, M_j^1\}$, where $M_y^0 + M_y^1 = I$ for $y = 0, 1, 2$. Since $\mathcal{W} = \sum_{x,y}(-1)^{x_y}P(0|x, y)$ is the linear expression of the probabilities, we can only consider the pure states in the numerical calculation.

$$\begin{aligned}\mathcal{W} &= \sum_{x,y}(-1)^{x_y}P(0|x,y) = \sum_{x,y}(-1)^{x_y}tr(\rho_x M_y^0)\\ &= \frac{1}{2}[(\overrightarrow{a_0} + \overrightarrow{a_1} + \overrightarrow{a_2})\cdot(\overrightarrow{r_{000}} - \overrightarrow{r_{111}}) + (\overrightarrow{a_0} + \overrightarrow{a_1} - \overrightarrow{a_2})\cdot(\overrightarrow{r_{001}} - \overrightarrow{r_{110}}) + (\overrightarrow{a_0} - \overrightarrow{a_1} + \overrightarrow{a_1})\cdot(\overrightarrow{r_{010}} - \overrightarrow{r_{101}})\\ &\quad + (\overrightarrow{a_0} - \overrightarrow{a_1} - \overrightarrow{a_1})\cdot(\overrightarrow{r_{011}} - \overrightarrow{r_{100}})],\end{aligned} \tag{7}$$

where $\overrightarrow{r_x}, \overrightarrow{a_y}$ represent the Bloch vectors of the pure states ρ_x and the projective measurement operators M_y^0, respectively.

Suppose that the projections of $\overrightarrow{a_2}$, $\overrightarrow{r_{000}}$ are $\overrightarrow{a_2}'$ and $\overrightarrow{r_{000}}'$ in the plane, which is spanned by the vectors $\overrightarrow{a_0}$ and $\overrightarrow{a_1}$. Let $\alpha, \alpha_1, \beta, \beta_1, \theta$ be the angles between $\overrightarrow{a_2}'$ and $\overrightarrow{a_0} + \overrightarrow{a_1}$, $\overrightarrow{r_{000}}'$ and $\overrightarrow{a_0}$, $\overrightarrow{a_2}$ and $\overrightarrow{a_2}'$, $\overrightarrow{r_{000}}$ and $\overrightarrow{r_{000}}'$, $\overrightarrow{a_0}$ and $\overrightarrow{a_1}$, respectively. We get

$$\overrightarrow{a_0}\cdot\overrightarrow{r_{000}} = \cos\beta_1\cos\alpha_1, \quad \overrightarrow{a_1}\cdot\overrightarrow{r_{000}} = \cos\beta_1\cos(\theta - \alpha_1), \tag{8}$$

$$\overrightarrow{a_2}\cdot\overrightarrow{r_{000}} = \cos(\beta - \beta_1)\cos(\frac{\theta}{2} - \alpha_1 - \alpha). \tag{9}$$

Let $\gamma_1, \gamma_2, \gamma_3$ be the angle between $\overrightarrow{r_{001}}$ and $\overrightarrow{r_{110}}$, $\overrightarrow{r_{010}}$ and $\overrightarrow{r_{101}}$, $\overrightarrow{r_{011}}$ and $\overrightarrow{r_{100}}$, respectively. We get

$$\overrightarrow{r_{001}} - \overrightarrow{r_{110}} = 2\sin\frac{\gamma_1}{2}\overrightarrow{x_1}, \quad \overrightarrow{r_{010}} - \overrightarrow{r_{101}} = 2\sin\frac{\gamma_2}{2}\overrightarrow{x_2}, \quad \overrightarrow{r_{011}} - \overrightarrow{r_{100}} = 2\sin\frac{\gamma_3}{2}\overrightarrow{x_3}, \tag{10}$$

$$\overrightarrow{v_1} = \overrightarrow{a_0} + \overrightarrow{a_1} = 2\cos\frac{\theta}{2}\overrightarrow{y_1}, \quad \overrightarrow{v_2} = \overrightarrow{a_0} - \overrightarrow{a_1} = 2\sin\frac{\theta}{2}\overrightarrow{y_2}, \tag{11}$$

$$\overrightarrow{a_0} + \overrightarrow{a_1} + \overrightarrow{a_2} = \overrightarrow{v_1} + \overrightarrow{a_2} = \sqrt{4\cos^2\frac{\theta}{2} + 1 + 4\cos\frac{\theta}{2}\cos\beta\cos\alpha}\,\overrightarrow{z_1}, \tag{12}$$

$$\overrightarrow{a_0} + \overrightarrow{a_1} - \overrightarrow{a_2} = \overrightarrow{v_1} - \overrightarrow{a_2} = \sqrt{4\cos^2\frac{\theta}{2} + 1 - 4\cos\frac{\theta}{2}\cos\beta\cos\alpha}\,\overrightarrow{z_2}, \tag{13}$$

$$\overrightarrow{a_0} - \overrightarrow{a_1} + \overrightarrow{a_2} = \overrightarrow{v_2} + \overrightarrow{a_2} = \sqrt{4\sin^2\frac{\theta}{2} + 1 + 4\sin\frac{\theta}{2}\cos\beta\sin\alpha}\,\overrightarrow{z_3}, \tag{14}$$

$$\overrightarrow{a_0} - \overrightarrow{a_1} - \overrightarrow{a_2} = \overrightarrow{v_2} - \overrightarrow{a_2} = \sqrt{4\sin^2\frac{\theta}{2} + 1 - 4\sin\frac{\theta}{2}\cos\beta\sin\alpha}\,\overrightarrow{z_4}, \tag{15}$$

where $\overrightarrow{x_1}$ represents the unit vectors overlapped with $\overrightarrow{r_{001}} - \overrightarrow{r_{110}}$, and others ($\overrightarrow{x_i}, \overrightarrow{y_i}, \overrightarrow{z_i}(i \in \{1,2,3,4\})$) have the similar definitions.

Further, by applying equations (9)-(15), equation(7) can be described as

$$\begin{aligned}\mathcal{W} =& \frac{1}{2}[\cos\beta_1\cos\alpha_1 + \cos\beta_1\cos(\theta - \alpha_1) + \cos(\beta - \beta_1)\cos(\frac{\theta}{2} - \alpha - \alpha_1)\\ &+\sqrt{4\cos^2\frac{\theta}{2} + 1 + 4\cos\frac{\theta}{2}\cos\beta\cos\alpha}\,\overrightarrow{z_1}\cdot(-\overrightarrow{r_{111}})\end{aligned} \tag{16}$$

$$
\begin{aligned}
&+\sqrt{4\cos^2\frac{\theta}{2}+1-4\cos\frac{\theta}{2}\cos\beta\cos\alpha}\,\overrightarrow{z_2}\cdot 2\sin\frac{\gamma_1}{2}\overrightarrow{x_1}\\
&+\sqrt{4\sin^2\frac{\theta}{2}+1+4\sin\frac{\theta}{2}\cos\beta\sin\alpha}\,\overrightarrow{z_3}\cdot 2\sin\frac{\gamma_2}{2}\overrightarrow{x_2}\\
&+\sqrt{4\sin^2\frac{\theta}{2}+1-4\sin\frac{\theta}{2}\cos\beta\sin\alpha}\,\overrightarrow{z_4}\cdot 2\sin\frac{\gamma_3}{2}\overrightarrow{x_3}].
\end{aligned}
\tag{17}
$$

Without loss of generality, suppose that the angle between $\overrightarrow{r_{000}}$ and $\overrightarrow{a_0}$ is the minimum, which satisfy that the guessing probability $p(0|000,0)$ is the maximum. We obtain that $\alpha_1 \in [0, \frac{\pi}{4}]$, $\theta \in [2\alpha_1, \pi-2\alpha_1]$, $\beta \in [0, \frac{\pi}{2}]$, $\alpha \in [\frac{\pi}{2}, \pi]$, $\cos\beta_1 \geq -\frac{\sqrt{[\cos\alpha_1-\cos\beta\cos(\frac{\theta}{2}-\alpha-\alpha_1)]^2+\sin^2\beta}}{\cos(\frac{\theta}{2}-\alpha-\alpha_1)\sin\beta}$. Fixing the angles of α_1, β_1, θ and applying equation (17), we get the maximal value of $\mathcal{W}$ expression

$$
\begin{aligned}
\mathcal{W}_{\alpha_1,\beta_1,\theta} =&\frac{1}{2}[\cos\beta_1\cos\alpha_1+\cos\beta_1\cos(\theta-\alpha_1)+\cos(\beta-\beta_1)\cos(\frac{\theta}{2}-\alpha-\alpha_1)]\\
&+\frac{1}{2}\sqrt{4\cos^2\frac{\theta}{2}+1+4\cos\frac{\theta}{2}\cos\beta\cos\alpha}\\
&+\sqrt{4\cos^2\frac{\theta}{2}+1-4\cos\frac{\theta}{2}\cos\beta\cos\alpha}\\
&+\sqrt{4\sin^2\frac{\theta}{2}+1+4\sin\frac{\theta}{2}\cos\beta\sin\alpha}\\
&+\sqrt{4\sin^2\frac{\theta}{2}+1-4\sin\frac{\theta}{2}\cos\beta\sin\alpha},
\end{aligned}
\tag{18}
$$

where the first equality holds because of choosing $\overrightarrow{z_1}=-\overrightarrow{r_{111}}, \gamma_1=\gamma_2=\gamma_3=\pi, \overrightarrow{x_i}=\overrightarrow{z_{i+1}}(i\in\{1,2,3\})$.

In order to obtain the maximal value of $\mathcal{W}$ expression only about the maximal guessing probability $\mathbf{p}=\frac{1+\cos\beta_1\cos\alpha_1}{2}$, we use the method of the extreme-value problem of multi-variable function and let $x=\cos\frac{\theta}{2}$, $y=\cos\beta$, $z=\cos\alpha$ and $u=\cos\alpha_1$ to apply to equation (18). Then we get

$$
\begin{aligned}
\mathcal{W}_{\mathbf{p}}^{\max} =&\max_{\{(r,s,v,m)\}}\{(2\mathbf{p}-1)r^2+(2\mathbf{p}-1)\frac{\sqrt{1-m^2}}{m}r\sqrt{1-r^2}+\frac{(2\mathbf{p}-1)s+\sqrt{1-s^2}\sqrt{m^2-(2\mathbf{p}-1)^2}}{2m}\\
&[(rv+\sqrt{1-r^2}\sqrt{1-v^2})m+(\sqrt{1-r^2}v-r\sqrt{1-v^2})\sqrt{1-m^2}]+\frac{1}{2}\sqrt{4r^2+1+4rsv}\\
&+\sqrt{4r^2+1-4rsv}+\sqrt{4(1-r^2)+1+4\sqrt{1-r^2}s\sqrt{1-v^2}}+\sqrt{4(1-r^2)+1-4\sqrt{1-r^2}s\sqrt{1-v^2}}\},
\end{aligned}
\tag{19}
$$

where (r,s,v,m) is one of the real roots of equation set with variables (x,y,z,u) in the following.

$$
\begin{aligned}
&-2(2\mathbf{p}-1)\sqrt{u^2-(2\mathbf{p}-1)^2}x\sqrt{1-x^2}+u^3\sqrt{1-u^2}\sqrt{1-y^2}(xz+\sqrt{1-x^2})+[u^2\sqrt{1-u^2}\sqrt{1-y^2}\\
&\quad-(2\mathbf{p}-1)y\sqrt{u^2-(2\mathbf{p}-1)^2}-\sqrt{1-y^2}(u^2-(2\mathbf{p}-1)^2)](\sqrt{1-x^2}z-x\sqrt{1-z^2})=0,
\end{aligned}
\tag{20}
$$

$$
\begin{aligned}
&[(2\mathbf{p}-1)\sqrt{1-y^2}-y\sqrt{u^2-(2\mathbf{p}-1)^2}][(xz+\sqrt{1-x^2}\sqrt{1-z^2})u+(\sqrt{1-x^2}z-x\sqrt{1-z^2})\sqrt{1-u^2}]\\
&\quad ABCD+2u\sqrt{1-y^2}xzCD(B-2A)+4u\sqrt{1-x^2}\sqrt{1-y^2}\sqrt{1-z^2}AB(D-C)=0,
\end{aligned}
\tag{21}
$$

$$
\begin{aligned}
&[(2\mathbf{p}-1)y+\sqrt{1-y^2}\sqrt{u^2-(2\mathbf{p}-1)^2}][(xz+\sqrt{1-x^2}\sqrt{1-z^2})\sqrt{1-u^2}-(\sqrt{1-x^2}z-x\sqrt{1-z^2})u]\\
&\quad ABCD+2uxy\sqrt{1-z^2}CD(B-2A)-4u\sqrt{1-x^2}yzAB(D-C)=0,
\end{aligned}
\tag{22}
$$

$$
\begin{aligned}
&\{4u(2\mathbf{p}-1)x\sqrt{1-x^2}+2(2\mathbf{p}-1)\sqrt{1-u^2}(1-2x^2)+[(2\mathbf{p}-1)y+\sqrt{1-y^2}\sqrt{u^2-(2\mathbf{p}-1)^2}][(\sqrt{1-x^2}z\\
&-x\sqrt{1-z^2})u-(xz+\sqrt{1-x^2}\sqrt{1-z^2})\sqrt{1-u^2}]\}ABCD+2u(2x+yz)\sqrt{1-x^2}BCD+4u(2x-yz)\sqrt{1-x^2}ACD\\
&\quad-4u(2x\sqrt{1-x^2}+xy\sqrt{1-z^2})ABD-4u(2x\sqrt{1-x^2}-xy\sqrt{1-z^2})ABC=0,
\end{aligned}
\tag{23}
$$

where

$$A = \sqrt{4x^2 + 1 + 4xyz}, \quad B = \sqrt{4x^2 + 1 - 4xyz}, \tag{24}$$

$$C = \sqrt{4(1-x^2) + 1 + 4\sqrt{1-x^2}y\sqrt{1-z^2}}, \tag{25}$$

$$D = \sqrt{4(1-x^2) + 1 - 4\sqrt{1-x^2}y\sqrt{1-z^2}}. \tag{26}$$

□

Analytical relation under the practical condition.

Proof of Lemma 3. Suppose that $\alpha_{\max} = \max\{\alpha_{b,x,y}\}$, $P_{\min} = \min\{P_X(x)P_Y(y)\}$, W_Q is the maximal value of $\mathcal{W}$ expression obeying quantum theory and t is the number of experiment's rounds.

Denote $\Delta_k[b^k, x^k, y^k] = \sum_{i=1}^{k}(\hat{\mathcal{W}}_i - \mathcal{W}_i[b^i, x^i, y^i])$, the fact $E(\hat{\mathcal{W}}_i|b^{i-1}, x^{i-1}, y^{i-1}) = \mathcal{W}_i[b^i, x^i, y^i]$ holds.

It is easy to verify

(i)

$$E(|\Delta_k|) = \sum_{i=1}^{k} E(|\hat{\mathcal{W}}_i - \mathcal{W}_i[b^i, x^i, y^i]|) \leq \sum_{i=1}^{k}(\frac{\alpha_{\max}}{P_{\min}} + W_{\max}) < \infty, \tag{27}$$

(ii)

$$E(\Delta_k|e^1, e^2, .., e^i) = E(\Delta_k|e^i) = \Delta_i, \tag{28}$$

for $i \leq k$.

Similar to the methods of the Refs. [3, 4], the sequence of random variables $\Delta_1, \Delta_2, ..., \Delta_t$ is a martingale [2] with respect to the sequence $e^1, e^2, ..., e^t$.

Using Azuma-Hoeffding inequality [2], we get

$$P(\Delta_t[b^t, x^t, y^t] \geq t\delta) \leq \exp(\frac{-(t\delta)^2}{2t\mu^2}), \tag{29}$$

where $\mu = \frac{\alpha_{\max}}{P_{\min}} + W_Q$. As we know, $\bar{\mathcal{W}} = \frac{1}{t}\{\sum_{i=1}^{t}\hat{\mathcal{W}}_i - \Delta_t[b^t, x^t, y^t]\}$, then

$$P(\bar{\mathcal{W}} \geq \hat{\mathcal{W}} - \delta) \geq 1 - 2^{\frac{-t\delta^2}{2ln2\mu^2}}. \tag{30}$$

□

Proof of Lemma 4. According to Lemma 3, we learn that

$$P(\overline{\mathcal{G}_1}) \leq 2^{\frac{-t\delta^2}{2ln2\mu^2}}, \tag{31}$$

where $\overline{\mathcal{G}_1}$ represents the complement event of $\mathcal{G}_1$, that is, $\overline{\mathcal{G}_1} = \{(b^t, x^t, y^t, l)|\bar{\mathcal{W}} \leq \hat{\mathcal{W}} - \delta\}$.

Based on $\mathcal{G}_2 = \{(b^t, x^t, y^t, l)|P(\mathcal{G}_1|x^t, y^t) \geq \frac{1}{2}\}$, then $P(\overline{\mathcal{G}_1}|\overline{\mathcal{G}_2}) \geq \frac{1}{2}$. Further,

$$P(\overline{\mathcal{G}_2}) = \frac{P(\overline{\mathcal{G}_1}, \overline{\mathcal{G}_2})}{P(\overline{\mathcal{G}_1}|\overline{\mathcal{G}_2})} \leq \frac{P(\overline{\mathcal{G}_1})}{P(\overline{\mathcal{G}_1}|\overline{\mathcal{G}_2})} \leq 2 \cdot 2^{\frac{-t\delta^2}{2ln2\mu^2}}. \tag{32}$$

According to $\mathcal{G}_3 = \{(b^t, x^t, y^t, l)|P_{L|X^tY^t\mathcal{G}_1}(l|x^t, y^t) \geq \frac{1}{\mathfrak{L}^2}\}$, then

$$P(\overline{\mathcal{G}_3}) = \sum_{(b^t, x^t, y^t, l) \notin \mathcal{G}_3} P_{X^tY^t|\mathcal{G}_1}(x^t, y^t) P_{L|X^tY^t\mathcal{G}_1}(l|x^t, y^t) \leq \frac{1}{\mathfrak{L}}. \tag{33}$$

It is known that $\mathcal{G} = \mathcal{G}_1 \cap \mathcal{G}_2 \cap \mathcal{G}_3$, and we get

$$\begin{aligned} P(\mathcal{G}) &= 1 - P(\overline{\mathcal{G}_1 \cap \mathcal{G}_2 \cap \mathcal{G}_3}) \\ &= 1 - P(\overline{\mathcal{G}_1} \cup \overline{\mathcal{G}_2} \cup \overline{\mathcal{G}_3}) \\ &\geq 1 - P(\overline{\mathcal{G}_1}) - P(\overline{\mathcal{G}_2}) - P(\overline{\mathcal{G}_3}) \\ &\geq 1 - 3 \cdot 2^{\frac{-t\delta^2}{2ln2\mu^2}} - \frac{1}{\mathfrak{L}}. \end{aligned} \tag{34}$$

□

Randomness extraction.

A set of functions $\mathfrak{F}$ from $\mathbf{B}^t$ to $\mathbf{R}^s$ is *two-universal* [5] if $f_s \in \mathfrak{F}$ is picked using a uniform random variable S. For any distinct instances, b_1^t and b_2^t of B^t, the probability that they give the same value of R^s is at most $\frac{1}{|\mathbf{R}|^s}$; that is,

$$P(f_s(b_1^t) = f_s(b_2^t)) \leq \frac{1}{|\mathbf{R}|^s}. \tag{35}$$

Proof of Lemma 6. We take the square of the left-hand side (2) and use the convexity of the square function, we obtain

$$\begin{aligned}
&[\sum_{r^{n_s},f} |P(r^{n_s}, f|x^t, y^t, l, \mathcal{G}) - 2^{-n_s} P(f|x^t, y^t, l, \mathcal{G})|]^2 \\
\leq & 2^{-n_s} \sum_{r,f} P(f|x^t, y^t, l, \mathcal{G})[2^{n_s} \sum_{b^t} P(b^t|x^t, y^t, l, f, \mathcal{G}) \delta_{f(b^t)}^{r^{n_s}} - 1]^2 \\
\leq & 2^{-n_s} \sum_{f} P(f|x^t, y^t, l, \mathcal{G})[2^{n_s} - 2^{1+n_s} + 2^{2n_s} \sum_{b^t, b'^t} P(b^t|x^t, y^t, l, f, \mathcal{G}) P(b'^t|x^t, y^t, l, f, \mathcal{G}) \delta_{f(b^t)}^{f(b'^t)}] \\
= & 2^{n_s} \sum_{f} P(f|x^t, y^t, l, \mathcal{G}) \sum_{b^t \neq b'^t} P(b^t|x^t, y^t, l, f, \mathcal{G}) P(b'^t|x^t, y^t, l, f, \mathcal{G}) \delta_{f(b^t)}^{f(b'^t)} \\
& + 2^{n_s} \sum_{f} P(f|x^t, y^t, l, \mathcal{G}) \sum_{b^t = b'^t} P^2(b^t|x^t, y^t, l, f, \mathcal{G}) - 1 \\
\leq & 2^{n_s} \mathbf{p}(b^t|x^t, y^t, l, f, \mathcal{G}) \\
= & 2^{n_s} \mathbf{p}(b^t|x^t, y^t, l, \mathcal{G}).
\end{aligned} \tag{36}$$

The last equality holds because of f independent of variables $x, y, l, \mathcal{G}$. The penultimate inequality holds based on the statement that $\sum P_i^2 \leq \max P_i$, where $P = \{P_i\}$ is a probability distribution. □

References

1. Masanes, L. Extremal quantum correlation for N parties with two dichotomic observables per site. e-print arXiv: quant-ph/0512100.
2. Grimmentt, G. & Stirzaker, D. Probability and Random variables *Oxford University Press, Oxford,* (2001).
3. Pironio, S. *et al*, Random numbers certified by Bell's theorem. *Nature (London)* **464**, 1021 (2010).
4. Pironio, S. & Massar, S. Security and composability of randomness expansion from Bell inequalities. *Phys. Rev. A* **87**, 012336 (2013).
5. Bennett, C. H., Brassard, G., Crepeau, C. & Maurer, U. M. Generalized privacy amplification. *IEEE Trans. Inf. Theory* **41**, 1915-1923, (1995).
